# Antimicrobial and Efflux Pump Inhibitory Activity of Carvotacetones from *Sphaeranthus africanus* Against Mycobacteria

**DOI:** 10.3390/antibiotics9070390

**Published:** 2020-07-08

**Authors:** Huyen Thi Tran, Julia Solnier, Eva-Maria Pferschy-Wenzig, Olaf Kunert, Liam Martin, Sanjib Bhakta, Loi Huynh, Tri Minh Le, Rudolf Bauer, Franz Bucar

**Affiliations:** 1 Institute of Pharmaceutical Sciences, Department of Pharmacognosy, University of Graz, Universitaetsplatz 4, 8010 Graz, Austria; tran.th.huyen@gmail.com (H.T.T.); julia.solnier@uni-graz.at (J.S.); eva-maria.wenzig@uni-graz.at (E.-M.P.-W.); rudolf.bauer@uni-graz.at (R.B.); 2 School of Medicine, Vietnam National University—HCMC, Quarter 6, Linh Trung Ward, Thu Duc District, HCM City 700000, Vietnam; leminhtri@uphcm.edu.vn; 3 Institute of Structural and Molecular Biology, Department of Biological Sciences, Birkbeck, University of London, Malet Street, London WC1E 7HX, UK; liam.tom.martin@gmail.com (L.M.); s.bhakta@bbk.ac.uk (S.B.); 4 Institute of Pharmaceutical Sciences, Department of Pharmaceutical Chemistry, University of Graz, Schubertstraße 1, 8010 Graz, Austria; olaf.kunert@uni-graz.at; 5 Department of Pharmacognosy, School of Medicine and Pharmacy, Da Nang University, 41 Le Duan Street, Hai Chau District, Da Nang City 550000, Vietnam; huynhloivn@gmail.com

**Keywords:** *Sphaeranthus africanus* L., Compositae, carvotacetones, mycobacteria, ethidium bromide accumulation, efflux pumps, efflux pump inhibitors

## Abstract

Carvotacetones (**1–7**) isolated from *Sphaeranthus africanus* were screened for their antimycobacterial and efflux pump (EP) inhibitory potential against the mycobacterial model strains *Mycobacterium smegmatis* mc^2^ 155, *Mycobacterium aurum* ATCC 23366, and *Mycobacterium bovis* BCG ATCC 35734. The minimum inhibitory concentrations (MICs) of the carvotacetones were detected through high-throughput spot culture growth inhibition (HT-SPOTi) and microbroth dilution assays. In order to assess the potential of the compounds **1** and **6** to accumulate ethidium bromide (EtBr) in *M. smegmatis* and *M. aurum*, a microtiter plate-based fluorometric assay was used to determine efflux activity. Compounds **1** and **6** were analyzed for their modulating effects on the MIC of EtBr and the antibiotic rifampicin (RIF) against *M. smegmatis*. Carvotacetones **1** and **6** had potent antibacterial effects on *M. aurum* and *M. bovis* BCG (MIC ≤ 31.25 mg/L) and could successfully enhance EtBr activity against *M. smegmatis*. Compound **1** appeared as the most efficient agent for impairing the efflux mechanism in *M. smegmatis*. Both compounds **1** and **6** were highly effective against *M. aurum* and *M. bovis* BCG. In particular, compound **1** was identified as a valuable candidate for inhibiting mycobacterial efflux mechanisms and as a promising adjuvant in the therapy of tuberculosis or other non-tubercular mycobacterial infections.

## 1. Introduction

Antimicrobial resistance (AMR) has developed against almost every known antibiotic [1]. Hence, substantial efforts in the discovery of innovative resistance-modifying agents are urgently required [2]. In recent years, the importance of further investments in this field of research and the production of new antibiotics to combat multidrug-resistant bacteria has been emphasized by the World Health Organization (WHO) [3]. Due to the alarming spread of bacterial resistance worldwide, therapeutic treatment options for infectious diseases, such as tuberculosis (TB), are becoming more and more limited [4,5]. According to a recent report from the WHO, TB remains one of the top ten causes of death worldwide and the leading cause from a single infectious agent [6]. Among the numerous self-defense strategies of bacteria in order to resist the toxic action of antibiotics, efflux pumps are one of the key tools for facilitating bacterial survival [7]. Efflux pumps are membrane transport proteins found in both Gram-positive and -negative bacteria, as well as in eukaryotes. By extruding noxious compounds out of the cells, efflux pumps reduce the intracellular concentration of antibiotics [8].

The administration of an efflux pump inhibitor (EPI) impairing the functionality of these pumps represents a promising approach to overcome bacterial multidrug resistance [4,9,10]. Recently, EPIs have gained particular attention as potent resistance-modifying agents capable of restoring the fading therapeutic efficacy of the available antibiotics, potentially facilitating their re-introduction into clinical use [10]. Although a variety of synthetic or plant-based EPIs have been characterized, no inhibitor has been clinically approved to date [11], due to pharmacological issues and/or toxic properties in vivo. As plants produce a diversity of biologically active compounds with a broad spectrum of pharmacological effects, they constitute a valuable resource for the discovery of potential EPIs [10]. Moreover, the combination of a natural EPI with an antibiotic drug may provide diverse clinical benefits in the treatment of infectious diseases [12].

Due to the large number of multidrug efflux pumps existing in mycobacteria, EPIs are of particular interest for the therapy of drug-resistant tuberculosis [13]. A distinctive feature of mycobacteria is their highly hydrophobic cell envelope, which presents a substantial permeability barrier rendering the bacteria resistant to many approved antibiotic classes [14]. *Mycobacterium smegmatis*, *Mycobacterium aurum*, and *Mycobacterium bovis* BCG represent useful surrogate models for tuberculosis-causing *Mycobacterium tuberculosis* and are therefore used in order to analyze efflux pump-mediated resistance, as well as for the in vitro screening of novel antimicrobial compounds [15]. Indeed, the anti-tubercular diarylquinoline drug candidate TMC207 was initially identified using *M. smegmatis* as a model organism [16]. Furthermore, the use of model strains, like *M. smegmatis* and *M. aurum*, accelerates the discovery of new antitubercular agents, while reducing the risk to researchers, and facilitating the screening of compounds in a non-category 3 bio-safety laboratory environment [4]. In addition, these fast-growing and environmental pathogenic organisms readily cause opportunistic infections in immunocompromised patients, categorized as non-tuberculous mycobacteria (NTM). NTM appear to be important human pathogens, since diverse infections caused by these bacteria have increased worldwide [17].

*Sphaeranthus*, a genus of the Compositae family, is known to contain compounds with anti-bacterial activity, as identified in crude extracts of *Sphaeranthus amaranthoides* Burm.f. [18] and *Sphaeranthus indicus* L. [19]. Two isolated carvotacetones and one mixture from *Sphaeranthus africanus* L. have been previously shown to possess antibacterial effects against *Escherichia coli*, *Pseudomonas aeruginosa*, and *Staphylococcus aureus*, but to be inactive against *Bacillus subtilis* [20].

Carvotacetones belong to the major volatile constituents of various genera within the Compositae family, such as *Blumea*, *Pulicaria*, and *Francoeuria*. In some essential oils of *Pluchea* and *Vernonia* species, carvotacetone was identified as the dominating constituent. Furthermore, carvotacetones could be detected in several other *Sphaeranthus* species showing anti-proliferative [21], anti-inflammatory [22], and anti-parasitic in vitro activities [23], as well as significant inhibitory effects on the ubiquitin–proteasome pathway [24]. 

**Figure 1:**
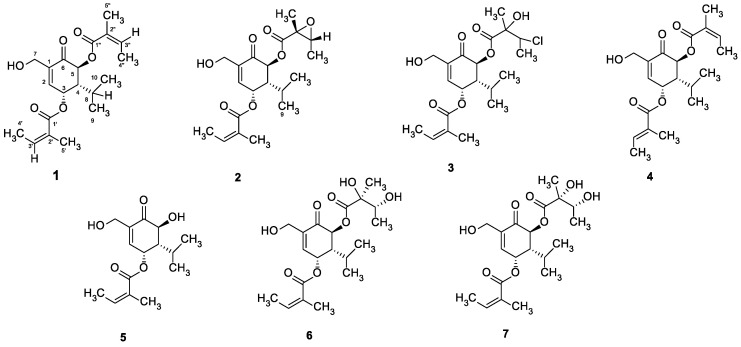
Chemical structures of compounds **1–7** isolated from *Sphaeranthus africanus* [21,22].

Compounds **1–7** (Figure 1) have been previously isolated from *Sphaeranthus africanus* L. and have shown anti-proliferative and anti-inflammatory activities in cellular models [21,22]. 

The aim of this study was to assess their potential antimicrobial and EPI effects in mycobacteria.

## 2. Results

### 2.1. Antimicrobial and Resistance Modulatory Activity

Seven compounds isolated from *Sphaeranthus africanus* were investigated for their antimicrobial effects, as well as for their potential to modulate the minimum inhibitory concentrations (MICs) of ethidium bromide (EtBr) and rifampicin against *M. smegmatis* mc^2^ 155, as demonstrated in Table 1. The antibacterial and MIC-modulating profile of compounds **1–7** towards *M. smegmatis*, a model strain expressing a variety of different efflux pumps, was determined via the use of microtiter broth dilution assays. In particular, the modulation assay with EtBr, which is a known substrate of efflux pumps, constitutes a valuable pre-screening method for plant compounds as potential EPIs [10].

The resistance modulatory activity of the compounds was determined at concentrations 0.25 × MIC. In modulation assays, the modulating factor (MF) illustrates putative adjuvant effects of the test substrates on the antimicrobial action of EtBr and rifampicin, a first-line antibiotic used for tuberculosis treatment. Out of seven compounds tested, compounds **1**, **3**, **4**, and **7** exhibited the strongest antibacterial effects against *M. smegmatis* mc^2^ 155 with MIC values ≤ 64 mg/L. Compounds **2**, **5**, and **6** exhibited only weak antimycobacterial activities at MICs of 128 mg/L. However, addition of **1** and **6** could significantly enhance the antimicrobial activity of EtBr against *M. smegmatis* leading to an eight-fold MIC reduction (MF_EtBr_ = 8). Furthermore, compound **1** affected the susceptibility of *M. smegmatis* towards rifampicin, lowering the rifampicin-resistance level 2-fold (MF_RIF_ = 2).

Due to the powerful modulating performance of compounds **1** and **6** reinforcing the antimicrobial activity of EtBr against *M. smegmatis* (MF = 8), these compounds were of particular interest for further investigations on *M. aurum* (ATCC23366) and *M. bovis* BCG (ATCC35734), and especially their EtBr-accumulation behavior in mycobacterial strains. 

HT-SPOTi, a high-throughput spot culture growth inhibition assay, served to assess the antimycobacterial profile of the substrates towards the bacterial strains *M. aurum* and *M. bovis* BCG. Table 2 presents the respective MICs of the tested compounds. It was found that both compounds **1** and **6** generated considerable antibacterial effects on the strains *M. aurum* and *M. bovis* BCG with MIC values ≤ 31.25 mg/L.

### 2.2. EtBr Accumulation

Accumulation experiments on the mycobacterial strains *M. smegmatis* and *M. aurum* were conducted with verapamil and chlorpromazine, two standard reference efflux pump inhibitors. EtBr is a favorable substance of efflux pumps and useful to study efflux activity, as it shows fluorescence when intercalating with hydrophobic regions, such as DNA, inside a bacterial cell [26]. 

In fact, the intracellular accumulation of EtBr enhanced by a putative EPI likely suggests a synergy between cell wall permeability and efflux inhibition, which can be measured through a fluorometric method [10,27]. Compounds **1** and **6** were analyzed for their potential to promote EtBr accumulation in *M. aurum* and *M. smegmatis*. Since efflux activity of EPIs in *M. bovis* BCG could not be explicitly determined, *M. aurum* and *M. smegmatis* were used as representative models for investigating EtBr accumulation in mycobacterial organisms. According to a study of Rodriguez et al. [28], only a basal efflux activity with EtBr took place, and the effects of EPIs in this strain were less clearly observable. This observation in *M. bovis* BCG is consistent with our experience (unpublished data). 

EtBr accumulation experiments were performed with substrates tested at MIC/2 in combination with glucose 0.4%, and EtBr used at a final concentration of 0.5 mg/L, leading to minimal EtBr accumulation. The conditions for the efflux experiments were chosen in order to facilitate the accumulation of EtBr, while not compromising the cell viability of the mycobacterial strains.

**Figure 2:**
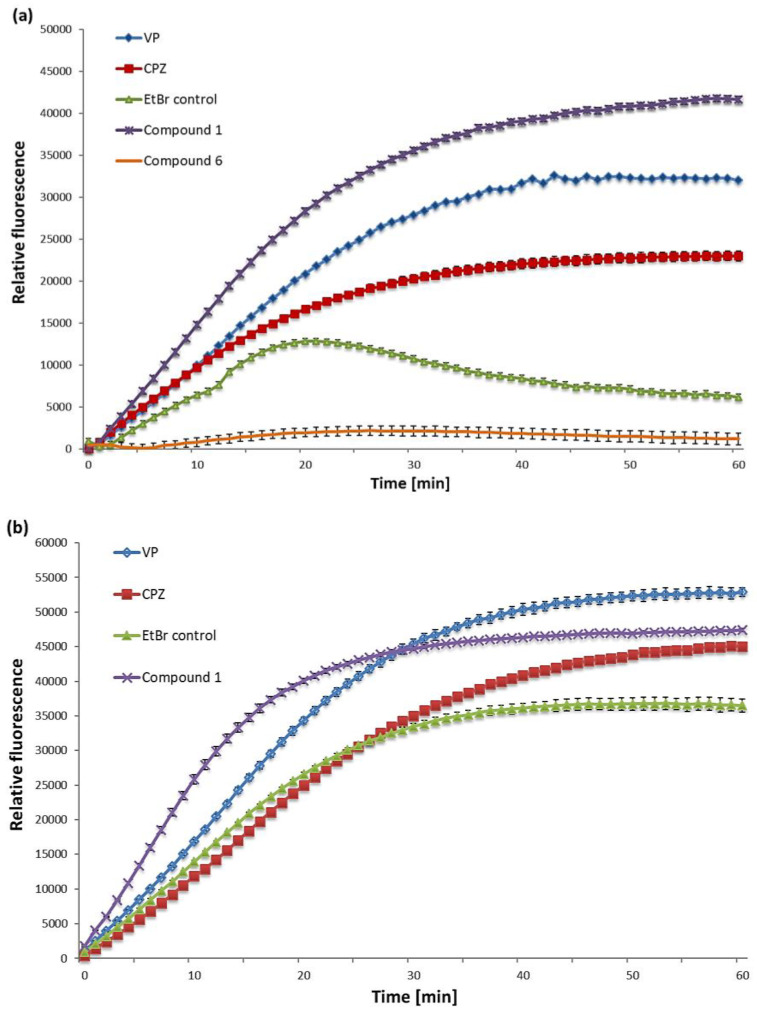
The increase of EtBr accumulation in *M*. *smegmatis* mc^2^ 155 (**a**) and *M. aurum* ATCC 23366 (**b**) generated by concentrations MIC/2 of the potential efflux pump inhibitors (EPIs) **1** and **6** and the reference inhibitors, verapamil (VP) and chlorpromazine (CPZ), included as two positive controls. EtBr, the negative control, was tested at 0.5 mg/L. The curves represent means ± standard deviations (SD); *n* = 3.

Compound **1** was found to be a very potent agent for blocking efflux of EtBr in *M. smegmatis* by generating the highest accumulation level in *M. smegmatis* when directly compared with the reference inhibitors verapamil and chlorpromazine (Figure 2a). In contrast, compound **6** could not trigger any accumulation of EtBr in the mycobacterial strain *M. smegmatis*. It might be suggested that compound **6** inhibited the intracellular accumulation of EtBr in *M. smegmatis* by affecting certain protein mechanisms, such as porin expression and cell wall permeability, or it may have interfered with the substance EtBr itself. 

In *M. aurum*, compound **1** induced greater accumulation of EtBr than chlorpromazine and had a similar impact on EtBr accumulation as verapamil; the detailed results are shown in Figure 2. More importantly, in this strain, compound **1** produced significant efflux inhibition activity within the first 20 minutes of the experiment by increasing the fluorescence of EtBr more rapidly than the reference inhibitors (Figure 2b). Due to a lack of material, compound **6** could not be further evaluated for EtBr accumulation in *M. aurum*.

## 3. Discussion

The antimycobacterial potential of some carvotacetones (**1–7**) from *Sphaeranthus africanus* L. was examined using the selected fast-growing mycobacterial strains *M. smegmatis* and *M. aurum*, and *M. bovis* BCG as a representative model organism for slow-growing mycobacteria. The two rapidly growing mycobacteria, *M. smegmatis* and *M. aurum*, allowed the screening of carvotacetones on efflux inhibition and modulatory activity. Studies on efflux mechanisms in *M. bovis* BCG were not performed, due to the slow-growing behavior of this strain.

Compounds **1–7** consist of a common basic scaffold, a 7-hydroxycarvotacetone, and differ in their C-3 and C-5 moieties, producing various derivatives. However, results of this study suggest that the composition of the acyl moiety at C-3 and C-5 of the respective compounds is crucial for antimycobacterial activity. Compounds **1** and **4** possess an angeloyl sidechain at C-5, which may be responsible for their greater activity against *M. smegmatis*. The distinctive sidechain at C-3 in compounds **1** (angeloyl group) and **4** (tiglolyl group) is likely responsible for the difference in their MIC modulating and EPI activity. Compound **1** had a significant modulating impact on EtBr activity (MF = 8) as well as EP inhibitory activity, in contrast to compound **4**, which was only weakly active. Compounds **6** and **7**, two diastereomeric compounds, had different antimicrobial efficiencies against *M. smegmatis*. In contrast to compound **7**, which showed strong antimicrobial activity against *M. smegmatis* (MIC = 64 mg/L), compound **6** only induced growth inhibition against *M. smegmatis* when tested in synergy with EtBr (MF = 8). Taken together, in contrast to compound **6**, compound **1** exhibited antimicrobial activity against all studied strains, together with a modulating effect on the efficiency of EtBr (MF_EtBr_ = 8) and rifampicin (MF_RIF_ = 2) against *M. smegmatis*.

Variations in the susceptibility profiles of the mycobacterial strains may be related to cell wall impermeability in association with different efflux pumps [27]. 

The genome of *M. smegmatis* contains several efflux genes, such as some MFS (major facilitator superfamily) transporters, different from those detected in *M. tuberculosis*, as well as other mycobacterial species [29]. Comparing the number of unique proteins reported for *M. smegmatis*, *M. aurum*, *M. bovis* BCG, and that for *M. tuberculosis*, showed that *M. smegmatis* contains a significantly higher number of 224 proteins as those found for the other mycobacterial strains [30]. 

According to the results presented in Table 2, the more lipophilic compound **1** obviously displays a greater antimicrobial efficiency on the tested mycobacterial strains than compound **6**, which has a more hydrophilic ester moiety bound to OH-5. Due to the typical lipid-rich cell envelope of mycobacteria, compounds of higher lipophilicities may induce stronger growth-inhibiting effects against the studied strains [31].

As regards efflux-pump inhibition, compound **1** could successfully achieve EtBr accumulation in both mycobacterial strains *M. smegmatis* and *M. aurum*, with greater effects than that of the reference inhibitors verapamil and chlorpromazine in *M. smegmatis*. Unlike compound **6**, showing no efflux-inhibitory activity, a link between potent modulating effects (MF_EtBr_ = 8) and efflux inhibitory activity of compound **1** might be suggested. However, the different incubation times of the bacterial cultures with the substrates including EtBr, used within the individual assays (72 h incubation for modulation assays; 1 h for accumulation assays), may not allow direct comparison of the results between these methods [4].

The impermeability of the lipophilic cell wall alongside efflux mechanisms contributes to the intracellular persistence of *M. tuberculosis* while negatively affecting the activity of established anti-TB drugs [32].

As a result of the present study, we propose specific lead compounds with a 7-hydroxycarvotacetone skeleton isolated from *Sphaeranthus africanus* as templates for potential new plant-derived antimicrobial agents and EPIs in order to optimize infection control and to tackle the problems related to rising multidrug resistance. The mycobacterial model strains *M. smegmatis* and *M. aurum* with gene encoding drug efflux pumps that are to some extent similar to those occurring in *M. tuberculosis* allowed us to investigate efflux-mediated resistance modulators in other mycobacteria including *M. tuberculosis*. Compound **1** from *Sphaeranthus africanus* appeared as a powerful antimicrobial agent with a considerable potentiating effect on the antibacterial activity of EtBr and of first-line anti-TB drug rifampicin against *M. smegmatis*. 

Within the scope of this research, we could identify compound **1** as a promising candidate for inhibiting mycobacterial efflux mechanisms. We believe that the mycobacterial strains used in this study serve as appropriate model organisms for investigating novel agents that are not only effective against tubercle bacilli, but also against non-tubercular opportunistic mycobacterial infections. 

In vitro studies are still justifiably the starting point for any drug design or discovery, and this original research study has paved the pathway for in vivo studies. Based on these findings, we propose compound **1** as a valuable candidate for further investigations.

## 4. Materials and Methods

### 4.1. Phytochemical Procedures

All detailed phytochemical and general experimental procedures on *S. africanus* L., such as isolations and structure identifications of the compounds **1–7** (Figure 1) are described in previously published research [21,22].

### 4.2. Plant Materials

*S. africanus* L. plant material, such as leaves and stems, were harvested in Vietnam, in Quảng Nam province. Genomic analysis was conducted by Prof. Dr. Günther Heubl, Faculty of Biology, Ludwig-Maximilians University, Munich, Germany. A voucher specimen (SA-VN-0216) was kindly provided for the University of Graz, Institute of Pharmaceutical Sciences, Department of Pharmacognosy.

### 4.3. Extractions and Isolations

A total of 1.5 kg of the air-dried, powdered (diameter 0.3–0.45 mm) leaves and stems of *S. africanus* were extracted with 20 L 96% ethanol at room temperature, resulting in 130 g of crude extract. After partitioning this crude extract with n-hexane, dichloromethane, ethyl acetate, and butanol, the following four sub-extracts were obtained: SA-Hex (6.0 g), SA-DCM (4.0 g), SA-Et (7.5 g), and SA-Bu (10 g), respectively. Sub-extracts were analyzed for phytochemical properties by using TLC, HPLC, and LC-MS. Carvotacetones **1–7** (Figure 1) were isolated from SA-DCM extract as described before [21,22]. A detailed description of the isolation procedure together with spectroscopic data used for the identification of structures **1–7** is presented in the reported literature [20,21].

### 4.4. Bacterial Strains and Culture Conditions

*Mycobacterium smegmatis* mc^2^ 155 (ATCC 700084, LCG Promochem, Teddington, Middlesex, UK) used for microbroth dilution experiments, such as MIC and modulation assays, was inoculated onto Columbia blood agar (Oxoid, Hampshire, UK) with supplementation of defibrinated horse blood 5% (Oxoid, Hampshire, UK) and was grown under aerobic conditions at 37 °C for 72 h. 

Slow-growing strain *Mycobacterium bovis* BCG ATCC 35734, and rapidly growing *Mycobacterium aurum* ATCC 23366 (purchased from the UK National Collection of Type Cultures), including *Mycobacterium smegmatis* mc^2^ 155, used for HT-SPOTi and/or EtBr accumulation experiments, were cultivated on Middlebrook 7H9 broth media enriched with OADC 10% (Sigma-Aldrich, St. Louis, MO, USA) under aerobic conditions. All bacterial cultures were stored in the Viabank system at -80 °C until required for testing.

### 4.5. MIC and Modulation Assays

Antimicrobial activity of *S. africanus* carvotacetones **1–7** (84–98% purity), including the antimicrobials rifampicin (Sigma-Aldrich) and isoniazid (Sigma-Aldrich), as well as EtBr (Sigma-Aldrich) against *M. smegmatis* mc^2^ 155 was evaluated by determining their minimum inhibitory concentrations (MIC) using a broth microdilution assay. Microbroth dilution assays were performed as described before by Gröblacher et. al [33]. Briefly, substrates were prepared in dimethyl sulfoxide (DMSO), and further diluted in Mueller–Hinton broth (MHB; Oxoid, Hampshire, UK). Isoniazid, used as positive control, along with a growth and sterile control, was integrated in each 96-well microtiter plate. Two-fold serial dilutions of the tested substances were carried out. A bacterial suspension was prepared in MHB to reach 5 × 10^5^ CFU/mL adjusted to McFarland 0.5 turbidity standard. Plates were incubated in aerobic conditions at 37 °C for 72 h. The MIC was determined by the addition of MTT solution (3-(4,5-dimethylthiazol-2-yl)-2,5-diphenyltetrazolium bromide in methanol; Sigma-Aldrich), measuring cell metabolic activity [34].

Spot culture growth inhibition (SPOTi) assay was conducted with *Mycobacterium bovis* BCG and *Mycobacterium aurum*, following the protocol established by Evangelopoulos and Bhakta, 2010 [35]. The MIC of a test substance was set as the minimum concentration required to inhibit the (visible) growth of mycobacterial organisms.

The resistance modulatory activity of the carvotacetones **1–7** isolated from *S. africanus* was determined through the performance of broth microdilution method. The microbroth dilution assay was carried out according to the method of Gröblacher et al. [33]. The substrates were tested at concentrations 0.25 × MIC and screened for their modulating impact on the antimicrobial efficacy of EtBr and the antitubercular drug rifampicin (RIF) against *M. smegmatis* mc^2^ 155. For the assessment of resistance modulation of the tested compounds, the modulation factor (MF) was determined according to the following formula. Significance of resistance modulation was defined at MF > 2.

MF = (MIC _antibiotic_)/(MIC _antibiotic+modulating compound_)

### 4.6. EtBr Accumulation Assay

To investigate the potential of the substrates **1** and **6** from *S. africanus* to accumulate EtBr in *M. smegmatis mc^2^* 155 and *M. aurum*, efflux assays were performed in accordance with the method described in more detail by Rodrigues et al [36].

Test substances **1** and **6**, including the reference inhibitors verapamil and chlorpromazine (Sigma Aldrich), were tested at MIC/2 for efflux experiments. The mycobacterial organisms *Mycobacterium aurum* and *Mycobacterium smegmatis* were cultivated in 10 mL of Middlebrook 7H9 broth containing 10% OADC enrichment at a temperature of 37 °C in aerobic conditions. Bacterial strains were diluted in Middlebrook 7H9 broth until a final optical density OD_600_ of 0.4 was achieved. After centrifugation of the mycobacterial suspension (10 min at 1008× *g*), and removal of the supernatant, the pellet was washed with 10 mL of PBS plus 0.05% Tween 80 (Sigma-Aldrich). The cultures and test substrates were combined in microtubes as working solutions. Blank solutions were prepared containing test substrates mixed with PBS plus 0.05% Tween 80, without bacterial cultures.

Test solutions were transferred into 96-well microtiter plates and EtBr, at a final concentration of 0.5 mg/L, was included in each well. The fluorescence of EtBr accumulation in *Mycobacterium aurum* and *Mycobacterium smegmatis* was measured by a fluorimeter (FLUOstar OPTIMA, BMG Labtech, Ortenberg, Germany), with parameters set at 544 nm excitation and 590 nm emission wavelengths at 37 °C in one-minute intervals for one hour. The reference inhibitors, verapamil and chlorpromazine used as positive controls, were included into EtBr accumulation experiments, and tested in the same procedure as described before.

## Figures and Tables

**Table 1:** Antimicrobial and resistance-modulating activity of the substrates against *M. smegmatis* mc^2^ 155 detected by microbroth dilution methods.

Compound	MIC		MIC [c]Modulator+EtBr	MIC [c]Modulator+RIF	MF (EtBr)	MF (RIF)
	(mg/L)	(μM)	(mg/L)	(mg/L)		
1	32	87.9	1	16	8	2
2	128	336.8	4	-	2	-
3	64	153.8	4	-	2	-
4	32	87.9	4	-	2	-
5	128	453.9	8	-	1	-
6	128	321.6	1	32	8	1
7	64	160.8	4	-	2	-
EtBr	8	20.3				
RIF	32	38.9				

Antimicrobial activity as the minimum inhibitory concentration (MIC). [c]Modulator = concentration MIC/4 used for the modulating compound. Determination of the modulating factor (MF) = [MIC_EtBr or antibiotic_/MIC_EtBr or antibiotic+Modulator_]; (-): not tested; *n* = 4.

**Table 2:** MIC values of the substrates against *M. smegmatis* mc^2^ 155 detected via microbroth dilution method. Spot culture growth inhibition (SPOTi) methods applied for testing with the strains *M. aurum* and *M. bovis* BCG.

Substrate	MIC					
	*M. smegmatis* mc^2^ 155		*M. aurum* ATCC 23366		*M. bovis* BCG ATCC 35734	
	(mg/L)	(μM)	(mg/L)	(μM)	(mg/L)	(μM)
1	32	87.9	31.25	85.9	15.63	42.9
6	128	321.6	31.25	78.5	31.25	78.5
Isoniazid	4	29.2	0.5	3.6	0.1	0.7
Verapamil	250	550.7	250	550.7	320 [25]	703.9
Chlorpromazine	25	78.6	20	62.9	20 [25]	62.8
EtBr	8	20.3	1	2.5	0.5 [25]	1.3

Antimicrobial activity as the minimum inhibitory concentration (MIC); *n* = 4.
